# Lipome cervical géant : À propos d'un cas à Ouagadougou, Burkina Faso

**DOI:** 10.48327/mtsi.v2i2.2022.234

**Published:** 2022-05-19

**Authors:** Moussa KADYOGO, Dina Alizèta COMPAORÉ, Aida Sandrine OUÉDRAOGO, Joséphine OUOBA, Donald BAYALA, Christine N. MEDA, Aimé Sosthène OUÉDRAOGO, Moustapha SEREME

**Affiliations:** 1 Service d'oto-rhino-laryngologie et de chirurgie cervico-faciale du Centre hospitalier universitaire de Bogodogo, Ouagadougou, Burkina Faso; 2 Service d'oto-rhino-laryngologie et de chirurgie cervico-faciale du Centre hospitalier universitaire pédiatrique Charles de Gaulle, Ouagadougou, Burkina Faso; 3 Service d'anatomie et de cytologie pathologiques du Centre hospitalier universitaire de Bogodogo, Ouagadougou, Burkina Faso; 4 Service de radiodiagnostic et d'imagerie médicale du Centre hospitalier universitaire de Bogodogo, Ouagadougou, Burkina Faso

**Keywords:** Lipome cervical géant, hôpital, Ouagadougou, Burkina Faso, Afrique subsaharienne, Giant cervical lipoma, hospital, Ouagadougou, Burkina Faso, Sub-Saharan Africa

## Abstract

**Objectif:**

Rapporter un cas rare de lipome cervical géant.

**Patients et méthodes:**

Il s'est agi d'un patient de 60 ans reçu en mars 2020 pour une tuméfaction cervicale antérieure progressive évoluant depuis 20 ans. Cette masse impactait négativement sur sa qualité de vie avec lourdeur cervicale, inconfort, limitation des mouvements de la tête, mais sans aucun signe de compression.

**Résultats:**

L'examen physique retrouvait une volumineuse masse cervicale paramédiane gauche de consistance ferme, sensible, mobile par rapport aux deux plans. La masse mesurait 13 cm de grand axe avec une peau en regard présentant des scarifications. Une cervicotomie a été réalisée sous anesthésie générale avec exérèse en bloc d'une masse encapsulée. L’évolution a été favorable avec une bonne cicatrisation. L'examen anatomopathologique de la pièce opératoire a conclu à un lipome bien différencié. Il n'y a pas eu de récidive après un recul de 24 mois.

**Conclusion:**

Le lipome cervical géant est rare. Son traitement est exclusivement chirurgical et l'examen histologique de la pièce opératoire confirme le diagnostic. Une surveillance postopératoire prolongée est recommandée en raison des risques de récidive et de dégénérescence maligne.

## Abstract (en)

**Objective:**

To report on a case of rare giant anterior cervical lipoma.

**Patients and methods:**

This was a 60-year-old male patient received in March 2020 for an anterior cervical swelling in progressive evolution since 20 years. The condition was negatively impacting the patient's quality of life with a feeling of cervical heaviness, discomfort, head movement limitations, with no sign of compression. Despite this significant discomfort, the patient first consulted several traditional practitioners with different traditional treatments without success, the mass having been taken for a goiter. It is in the face of the failure of traditherapeuts that the patient finally decided to consult in our care structure.

**Results:**

Physical examination found a large left paramedian formation which appeared soft, mobile vis-a-vis the two plans, and sensitive; it measured 13 cm on its longer axis, and surrounding skin showed scarifications, witness to previous traditional treatments. Cervical CT eliminated goiter and made it possible to diagnose a giant cervical lipoma. A cervicotomy was performed under general anesthesia with a total one-block excision of an encapsulated mass. The evolution was favorable with a good healing. The histological examination of the mass identified a well-differentiated lipoma. There was no relapse up until 24 months later.

**Conclusion:**

The giant anterior cervical lipoma is rare. The differential diagnosis is mainly posed with a large goiter or liposarcoma. CT or MRI make it possible to confirm the diagnosis. Its treatment is exclusively surgical and the examination of the operative piece by the pathologist confirms the diagnosis. An extended post-operative monitoring is recommended given the risks of relapse and of malignant degeneration.

## Introduction

Le lipome est une tumeur bénigne d'origine mésenchymateuse, généralement de petite taille mesurant moins de 5 cm. Il est qualifié de géant lorsque sa taille dépasse 10 cm ou lorsque son poids excède 1000 g [3, 4]. Le lipome représente 4 à 5 % de toutes les tumeurs bénignes des tissus mous avec une incidence annuelle estimée à un pour 1000 personnes. Ces tumeurs sont composées d'adipocytes matures [5].

Il peut survenir n'importe à n'importe quel endroit du corps où se trouve une accumulation de cellules graisseuses [2]. Environ 13 % des lipomes se développent au niveau de la tête et du cou [5]. Les lipomes du cou concernent en général la région cervicale postérieure. La localisation cervicale antérieure est rare et le lipome géant cervical antérieur est exceptionnel [2].

Nous rapportons un cas de lipome géant cervical antérieur pris en charge dans le service d'oto-rhino-laryngologie et de chirurgie cervico-faciale du Centre hospitalier universitaire de Bogodogo à Ouagadougou au Burkina Faso.

## Observation Clinique

**Figure 1:**
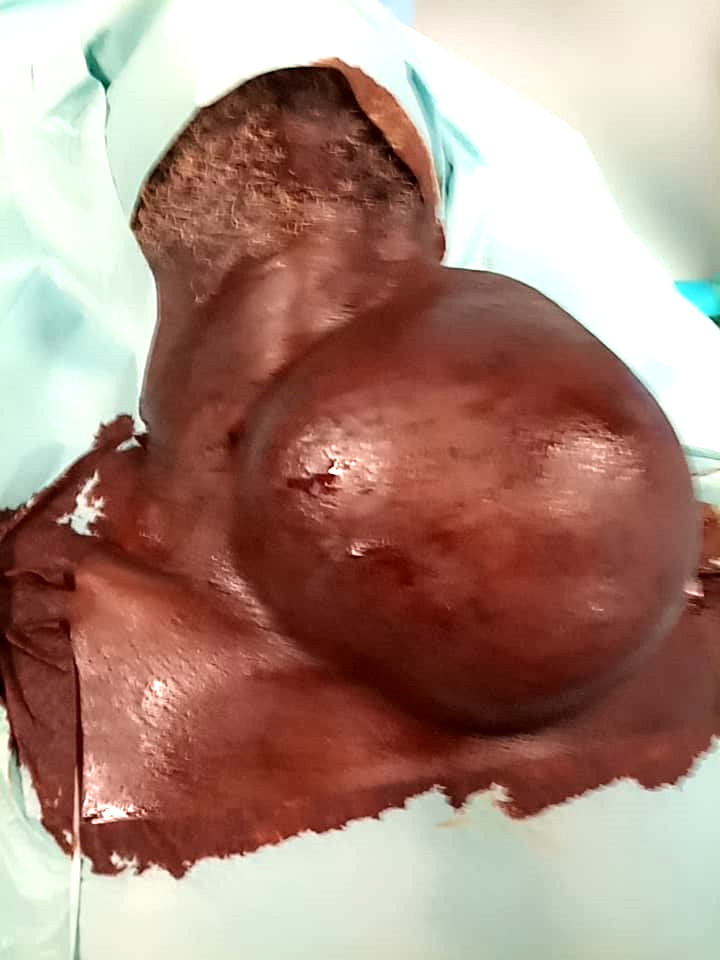
Lipome géant antéro-latéro-cervical gauche Giant left anterior cervical lipoma

Il s'agit d'un patient de 60 ans avec un antécédent de cure herniaire inguino-scrotale en novembre 2019, reçu en mars 2020 pour une masse cervicale antérieure évoluant depuis environ 20 ans (Fig. 1). Cette masse augmentait progressivement de volume et impactait négativement la qualité de vie du patient avec une sensation de lourdeur cervicale, d'inconfort et de limitation des mouvements de la tête, sans signe de compression. Il n'y avait pas de notion de maladie systémique telle qu'une infection par le VIH ou une maladie auto-immune. Le patient avait bénéficié d'un traitement traditionnel par des scarifications, mais sans résultat probant.

L'examen physique retrouvait une volumineuse masse cervicale antéro-paramédiane gauche, molle, mobile par rapport aux deux plans, sensible, mesurant 13 cm de grand axe avec une peau en regard présentant des scarifications. Il n'y avait pas d'adénopathie cervicale palpable.

**Figure 2:**
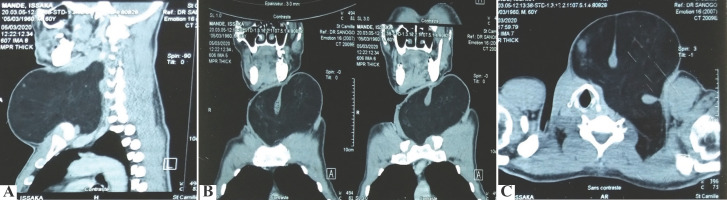
Tomodensitométrie cervico-thoracique : masse hypodense baso-antéro-latérale gauche bien limitée évoquant un lipome géant 2a : coupe sagittale 2b : coupe coronale 2c : coupe axiale Cervicothoracic CT scan: well-limited left anterolateral basal hypodense mass evoking a giant lipoma 2a: sagittal section 2b: coronal section 2c: axial section

L’échographie cervicale a mis en évidence une volumineuse masse cervico-thoracique hétérogène mal limitée d'au moins 800 cc de volume ne comprimant pas les gros vaisseaux. Un scanner cervico-thoracique objectivait une masse cervicale hypodense baso-antéro-latérale gauche d'allure graisseuse de 130/180 mm sans extension thoracique (Fig. 2a, 2b et 2c).

Une cervicotomie sous anesthésie générale a été réalisée avec exérèse en un seul bloc d'une masse encapsulée en totalité (Fig. 3a et 3b). Un drain de Redon a été placé, puis retiré 2 jours plus tard. Les suites opératoires ont été marquées à J7 par une tuméfaction fluctuante de la région antérieure du cou associée à une nécrose très limitée de la peau. Une ponction à l'aiguille a ramené environ 50 cc de sérosité. Le patient a bénéficié d'un décapage et d'un pansement quotidien. L’évolution a été favorable avec une cicatrisation complète.

**Figure 3a:**
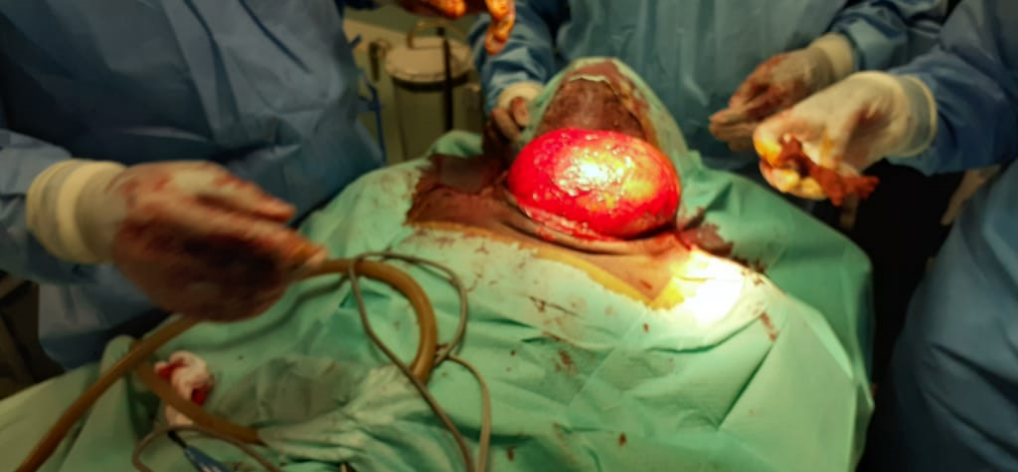
Vue opératoire, exposition du lipome Operative view of the lipoma

**Figure 3b:**
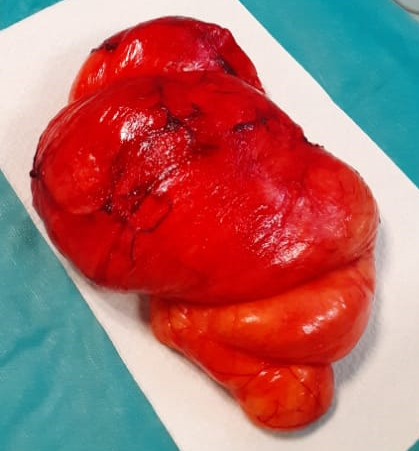
Pièce opératoire de lipome cervical géant Surgical specimen of the cervical giant lipoma

L'examen histologique concluait à un lipome bien différencié (Fig. 4a et 4b). Il n'y a pas eu de récidive avec un recul de 24 mois.

**Figure 4a:**
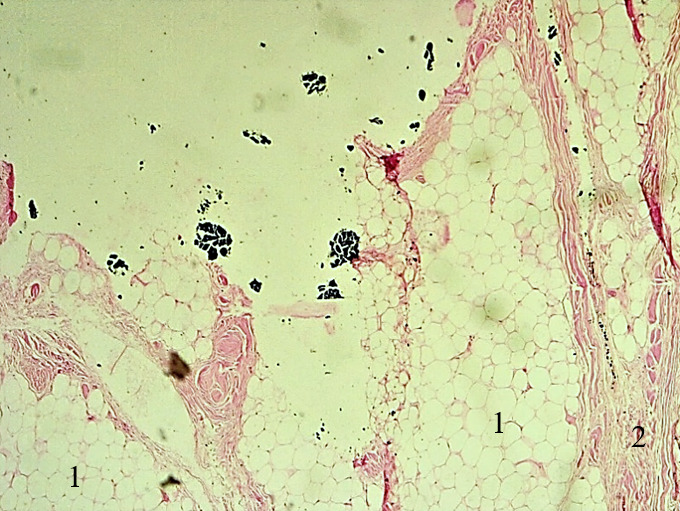
Lipome bien différencié; prolifération d'adipocytes matures groupés en lobules, séparés par de fins tractus fibreux, (HE, x40). 1 : lobules d'adipocytes matures; 2 : tractus fibreux Well-differentiated lipoma; proliferation of mature adipocytes grouped in lobules, separated by thin fibrous septa, (HE, x40). 1: mature adipocytes lobules; 2: fibrous tract

**Figure 4b:**
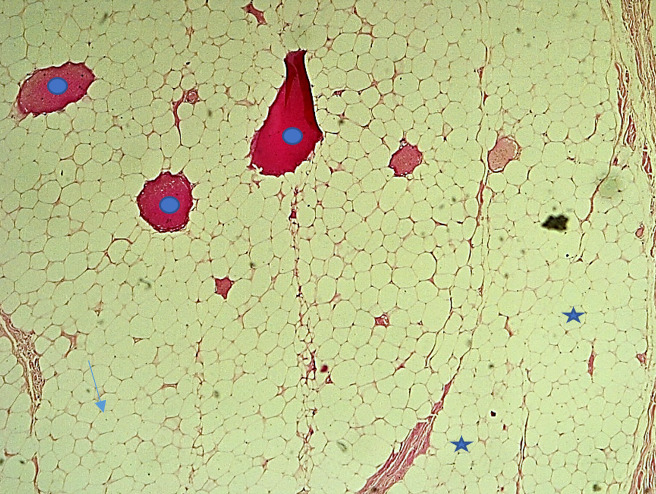
Adipocytes matures, cellules polyédriques optiquement vides au cytoplasme abondant avec de petits noyaux excentrés et aplatis sans atypies cytonucléaires (HE, x100). Étoile : adipocytes matures; Flèche : noyaux aplatis excentrés; Cercle : foyers de suffusion hémorragique Mature adipocytes, optically empty polyhedral cells with abundant cytoplasm and eccentrically pushed nuclei without cytonuclear atypia (HE, x100). Star: mature adipocytes; Arrow: eccentrically pushed nuclei; Circle: foci of hemorrhagic suffusion

## Discussion

Les lipomes solitaires sont les tumeurs des parties molles les plus fréquentes. Ce sont des tumeurs bénignes qui se développent aux dépens des adipocytes. Les lipomes sont le plus souvent encapsulés et de croissance lente. Ils peuvent être localisés dans n'importe quelle partie du corps. Cependant, les lipomes géants de localisation cervicale antérieure, comme celui présenté par notre patient, sont rares [1]. Ils surviennent surtout à l’âge adulte sans prédilection d'origine, ni de sexe.

Diarra *et al.* rapportaient dans leur étude un âge moyen de 51,16 ans et une prédominance masculine [1]. Notre patient était de sexe masculin, âgé de 60 ans. En général, le lipome cervical évolue sur plusieurs années. Une immunodépression acquise comme l'infection à VIH peut entraîner une croissance plus rapide du lipome [2, 7]. Par ailleurs, une augmentation rapide du volume de la masse devrait susciter des inquiétudes quant à sa transformation maligne [1].

L’étiologie et la pathogenèse du lipome restent inconnues, même si des facteurs génétiques et traumatiques ont été suggérés. Toutefois, le lipome survient le plus souvent chez les personnes en surpoids et augmente de volume quand le sujet prend du poids. Mais il n'a été établi aucune relation directe entre la perte de poids et la régression d'un lipome [5]. Aucun facteur de risque particulier n'a été retrouvé chez notre patient.

Les lipomes sont pour la plupart asymptomatiques. Néanmoins, certains cas de lipomes géants peuvent le devenir par compression des organes vitaux de voisinage. Ils posent en outre un handicap fonctionnel ou esthétique en raison de leur taille et de leur poids énormes [4, 5]. C’était le cas chez notre patient.

Son lipome a évolué progressivement sur une vingtaine d'années. La tuméfaction étant non douloureuse, elle n'a pas suscité d'inquiétude chez lui. Il n'a donc pas jugé nécessaire de consulter. Par ailleurs, ce genre de masses cervicales antérieures dans nos contrées passe pour des goitres. Et tant que le goitre ne se complique pas, les patients en général ne consultent pas un agent de santé. Beaucoup optent plutôt pour un traitement traditionnel. Les tradipraticiens vivent avec les communautés, ils sont plus proches des populations et inspirent une certaine confiance aux patients. En outre, la médecine traditionnelle coûte relativement moins cher que la médecine moderne.

Le diagnostic différentiel principal du lipome géant est le liposarcome. Ce dernier est bien limité mais non encapsulé et envahi les organes musculaires et osseux de voisinage [4, 5]. Lorsque la masse graisseuse augmente rapidement de volume et adhère aux structures de voisinage, elle doit susciter des inquiétudes quant à sa transformation maligne [4]. Le diagnostic différentiel peut aussi se poser avec un goitre.

Non seulement l'imagerie joue un rôle important dans le diagnostic de la nature graisseuse de la masse, mais elle permet de préciser son extension et ses rapports vasculo-nerveux [4]. Elle permet également de suspecter une éventuelle transformation maligne du lipome.

L’échographie reste l'examen de premier choix pour le diagnostic initial des masses cervicales. Elle montre une formation ovalaire ou allongée bien limitée à grand axe parallèle au plan cutané. Elle apparaît hypoéchogène ou hyperéchogène avec une structure homogène ou discrètement hétérogène [1, 5].

En tomodensitométrie (TDM), le lipome se présente comme une masse homogène hypointense, non rehaussée après injection de produit de contraste. Son signal est identique à la graisse sous cutanée. Sa densité est comprise entre environ -60 et -120 unités Hounsfield. En revanche, le goitre présente à la TDM une densité spontanée tissulaire (30-40 UH) qui est rehaussée après injection de produit de contraste.

En imagerie par résonance magnétique (IRM), le lipome se présente comme une lésion hyperintense en séquence T1, hypointense sur la séquence suppression du signal de la graisse. Le lipome ne prend pas de contraste après injection de gadolinium [4, 6]. En cas de suspicion de malignité (liposarcome), l'IRM est supérieure à la TDM pour le diagnostic compte tenu de sa haute performance pour les tissus mous [4]. En IRM, le liposarcome présente des cloisons épaisses et irrégulières et des plages nodulaires et tissulaires qui ressortent après injection de gadolinium.

Le diagnostic de certitude reste l'histologie, avec la mise en évidence d'adipocytes matures séparés par de fines cloisons et délimités par une pseudocapsule sans atypie cellulaire [4].

Le traitement du lipome géant cervical est chirurgical. Il consiste en une exérèse chirurgicale complète. Notre patient a consulté plusieurs tradithérapeutes, pensant qu'il s'agissait d'un goitre. Les différents traitements ont consisté en des scarifications au niveau de la peau en regard du lipome, avec application de médicaments traditionnels. C'est après l’échec du traitement traditionnel que le patient est enfin venu consulter dans notre structure sanitaire. Étant un commerçant aisé, il n'avait pas de difficulté financière particulière pour consulter un agent de santé ou se faire opérer. Il a plutôt laissé beaucoup d'argent chez les tradipraticiens avant d'avoir recours à la médecine moderne.

Une surveillance postopératoire régulière et prolongée est de mise en raison du risque de récidive et de transformation du lipome géant en liposarcome [4, 5].

## Conclusion

Le lipome géant cervical antérieur reste une entité clinique rare. L'imagerie joue un rôle primordial dans le diagnostic. Son traitement est exclusivement chirurgical, avec à la clé un examen anatomopathologique de la pièce opératoire qui confirmera le diagnostic. La surveillance post opératoire prolongée est de règle en raison des récidives possibles et du risque de dégénérescence maligne.

## Liens D'intérêts

Les auteurs ne déclarent ni lien, ni conflit d'intérêt. Cette étude a été réalisée à leurs propres frais. Ils n'ont reçu aucune contribution financière d'une tierce personne ou structure.

## Contribution Des Auteurs

Moussa KADYOGO : rédacteur principal du manuscrit, correspondant de l’étude

Dina Alizèta COMPAORE : a participé à la rédaction de l'article

Aida Sandrine OUEDRAOGO : a lu les lames et fourni l'iconographie en ce qui concerne l'anatomie pathologie

Joséphine OUOBA : a participé à l'intervention chirurgicale et à la rédaction du manuscrit

Donald BAYALA : a réalisé le scanner et fourni l'iconographie en ce qui concerne l'imagerie médicale (TDM)

Christine N. MEDA : a participé à la rédaction de l'article

Aimé Sosthène OUEDRAOGO : a lu les lames et fourni l'iconographie en ce qui concerne l'anatomie pathologie

Moustapha SEREME : a lu et corrigé le manuscrit
